# Horizontal mantle flow controls subduction dynamics

**DOI:** 10.1038/s41598-017-06551-y

**Published:** 2017-08-08

**Authors:** E. Ficini, L. Dal Zilio, C. Doglioni, T. V. Gerya

**Affiliations:** 1 Department of Earth Sciences, Sapienza University of Rome, Rome, Italy; 2 Institute of Geophysics, ETH Zurich, Zürich, Switzerland; 3 Istituto Nazionale di Geofisica e Vulcanologia, INGV, Rome, Italy

## Abstract

It is generally accepted that subduction is driven by downgoing-plate negative buoyancy. Yet plate age –the main control on buoyancy– exhibits little correlation with most of the present-day subduction velocities and slab dips. “West”-directed subduction zones are on average steeper (~65°) than “East”-directed (~27°). Also, a “westerly”-directed net rotation of the lithosphere relative to the mantle has been detected in the hotspot reference frame. Thus, the existence of an “easterly”-directed horizontal mantle wind could explain this subduction asymmetry, favouring steepening or lifting of slab dip angles. Here we test this hypothesis using high-resolution two-dimensional numerical thermomechanical models of oceanic plate subduction interacting with a mantle flow. Results show that when subduction polarity is opposite to that of the mantle flow, the descending slab dips subvertically and the hinge retreats, thus leading to the development of a back-arc basin. In contrast, concordance between mantle flow and subduction polarity results in shallow dipping subduction, hinge advance and pronounced topography of the overriding plate, regardless of their age-dependent negative buoyancy. Our results are consistent with seismicity data and tomographic images of subduction zones. Thus, our models may explain why subduction asymmetry is a common feature of convergent margins on Earth.

## Introduction

**Figure 1:**
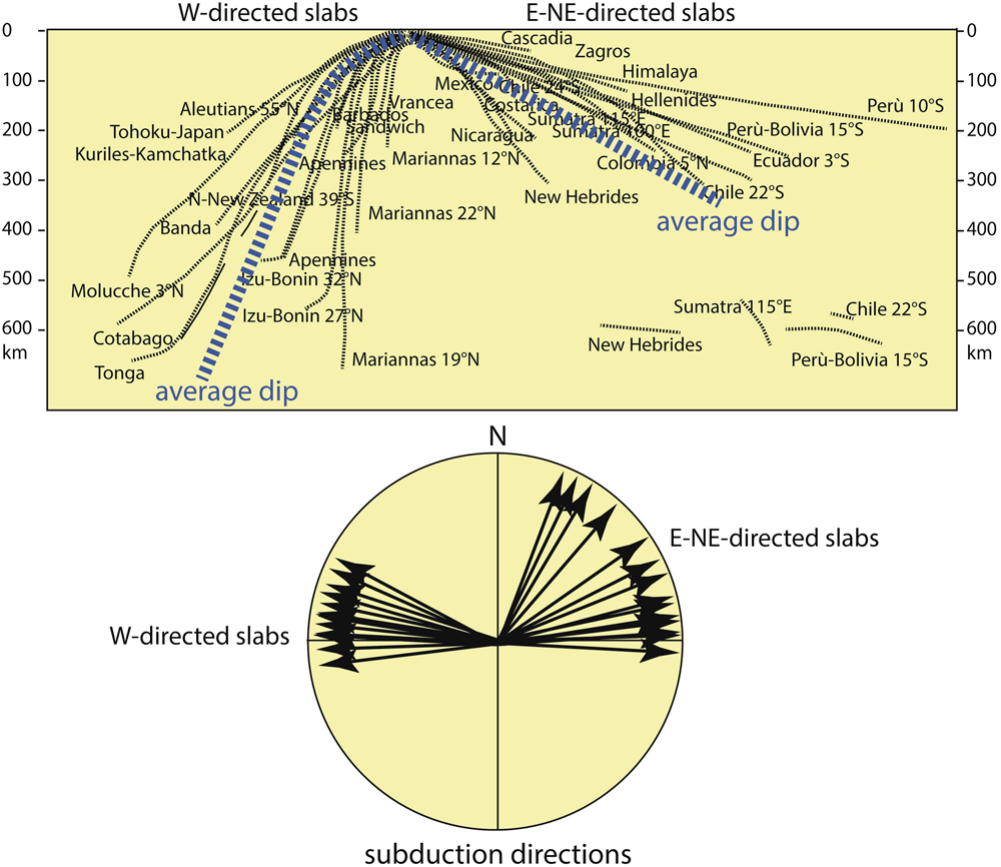
Slab dip of the main subduction zones of the world measured parallel to the convergence direction among upper and lower plates. Subduction directions appear concentrated into two main trends, i.e., W-ward and E-ward or N-NE-ward. W-directed subduction zones are steeper than E- or NE-ward directed subduction zones.

Several outstanding issues concerning subduction dynamics currently remain unaddressed^1–10^. For instance, it is not understood why most of today’s subducting plates interact differently with the mantle, which inevitably results in significant variations in slab morphology and dynamics worldwide^11, 12^. In particular, there is relevant evidence that terrestrial plate tectonics has a strongly asymmetric character, along both extensional^13^ and convergent plate boundaries^14^, which could be related to global asymmetry of lithosphere-mantle interactions moving along the so-called, undulated, tectonic equator^15^. When comparing “westward” versus “eastward” (or “northeastward”) directed subduction zones, there are systematic differences in their morphology, kinematics of the subduction hinge, gravity anomalies, heat flow, subsidence and uplift rates, etc. refs 12, 14, 16 and 17. The most remarkable feature that can be inferred from geophysical signatures is that W-directed slabs are generally very steep and deep (Fig. 1), they have a cogenetic back-arc basin, low-topography single-verging accretionary prism associated with them, and a very deep, fast subsiding (>1 mm/yr) foredeep basin or trench (e.g., Marianas). On the other hand, E- or NE- directed slabs are shallower (seismicity generally ends at about 300 km, apart from some deeper clusters close to the upper-lower mantle transition)^18^ and are less steep (Fig. 1); furthermore there is no typical back-arc basin opening but instead they build high-topography double-verging orogens with associated two shallower, slow subsiding (<0.1–0.3 mm/yr) trench or foreland basins^15^. These asymmetries are striking when comparing the western Pacific slabs and orogens (low topography and back-arc spreading in the upper plate) and the eastern Pacific subduction zones (high topography and deep rocks involved in the upper plate), which cannot be explained solely by variations in the age-dependent negative buoyancy of the subducting lithosphere^11, 15, 19, 20^. The same asymmetric features can be recognized worldwide, for instance, comparing the W-directed Atlantic subduction zones and the NE-directed Dinarides-Zagros-Himalayas-Indonesia subduction zones, and it seems rather to be related to the geographic polarity of subduction. In support of this hypothesis, geometrical asymmetries and geophysical constraints along subduction zones would point to a “westerly” polarized drift of plates^21^, which implies a relative opposed flow of the underlying Earth’s mantle (that is, easterly-directed “mantle wind”). This relative motion is presumably allowed by the presence of a rheologically weak sub-lithospheric low seismic velocity zone (LVZ) located at about 100–200 km depth, where low-degree (~1–2%) melting causes an abrupt drop in the velocity of seismic waves and a low viscosity of the asthenosphere (~10^17^–10^19^ Pa s)^22–24^.

The “westward” drift has been so far considered only from the perspective of an average motion of the lithosphere relative to the mantle, due to the larger weight of the Pacific plate in the hotspot reference frame^25–27^. This western residual of plate motion persists when plate kinematics are computed relative to Antarctica^28, 29^. There are two competing models on the “westerly” directed differential rotation of the lithosphere relative to the underlying mantle: (1) a slow mean net rotation (0.1–0.4°/Ma) in which few plates move eastward relative to the mantle (e.g., Nazca plate)^30, 31^, or (2) a fast complete lithospheric rotation (>1.2°/Ma), albeit at different speed of the individual plates, that is the so-called westward drift of the lithosphere with a pole of rotation located in the southeast Indian ocean at about 56° latitude and 136° longitude^21, 32^. There are also several hypothesis on the origin of the net rotation or the westward drift of the lithosphere such as lithosphere density and viscosity, lateral LVZ heterogeneities, etc. refs 11, 33–35.

The potential influence of westward lithospheric drift (or relative eastward mantle wind) on the subduction zones asymmetry has been already explored in the past decades on the basis of simplified approaches. For instance, an analytical model^1^ has been proposed to stress the importance of a background mantle flow in influencing slabs geometry. This model suggests that the dip angle of subducted slabs are strongly controlled by a large-scale flow imposed within the mantle by tectonic plates moving in their observed geometry and, more importantly, the slabs are orientated as if they were responding passively to the flow driven by the surface motion of the plates. Moreover, this model shows how important the decoupling role of a Low Viscosity Layer (LVZ) between lithosphere and mantle would be. In fact, the match between the direction of mantle flow and the direction of the subducted slab, given by the trend in earthquakes hypocentres, is good for most of the subduction zones and is usually improved by the inclusion of this decoupling level^1^. More recently, a purely mechanical physical model^6^ has been used to investigate influences of a regional convective (i.e., combined horizontal and vertical) mantle flow on the geometry of subducted slabs and the deformation regime of the overriding plates. It has been demonstrated that an imposed circulation of a simplified Newtonian asthenospheric mantle with rates of 1–10 cm yr^−1^ in the direction of subduction can cause flattening of slab angles from vertical to ∼60°. These modelling results have been applied to explain geophysical observations in some regional subduction settings (NE-Japan, Central America). Further numerical thermomechanical models^8^ have been used to understand how much of the slab-dip variability found in nature can be attributed to the interaction between the slab and a background mantle flow. The number of salient features of mantle-lithosphere interaction that these models do succeed in reproducing provides useful insights. However, these thermomechanical numerical models did not employ realistic free upper surface condition, which is crucial for subduction zones asymmetry^36^, and used highly simplified rheology of the mantle that is in conflict with available geochemical, experimental and theoretical data^37–39^. Hence, the validation of the mantle wind hypothesis, through a realistic state of the art numerical thermomechanical model, stands as a challenge that motivated our work.

Here, we explore and integrate the same effects of a priori defined mantle flow on the subduction zone morphology and slab dynamics, improving the modelling by means of self-consistent two-dimensional rheologically realistic thermomechanical numerical experiments with a free surface. In these experiments an oceanic plate sinks beneath a continental plate under the control of non-Newtonian temperature-, pressure- and strain rate-dependent viscous-plastic rheologies (with viscosity magnitude ranging from 10^18^ and 10^25^ - Supplementary Table S1) in a fully thermodynamically coupled model accounting for mineralogical phase changes^40^. A troughgoing^8^ purely horizontal (0–3 cm yr^−1^) asthenospheric mantle flow (i.e., not related to any regional mantle circulation)^6^ is imposed at both lateral boundaries in the same or opposite direction with respect to pre-defined rightward subduction polarity. We also tested the potential influence of the LVZ decoupling level (with a constant viscosity value of 10^18^ Pa s) on mantle-lithosphere interactions.

## Results

The use of mantle wind, in conjunction with a weak asthenospheric layer, produce sustained asymmetric subduction for the majority of models run in this study (see Supplementary Information Figs 5–10 and Table S2). The temporal evolution of two end-member models, one with discordant mantle flow and one with concordant mantle flow with respect to the subduction polarity, demonstrate the defining features of the evolution of all models. In the following sections we describe our end-member models, divided by direction of the mantle flow with respect to the subduction polarity.

### Models with discordant mantle flow and subduction polarity

**Figure 2:**
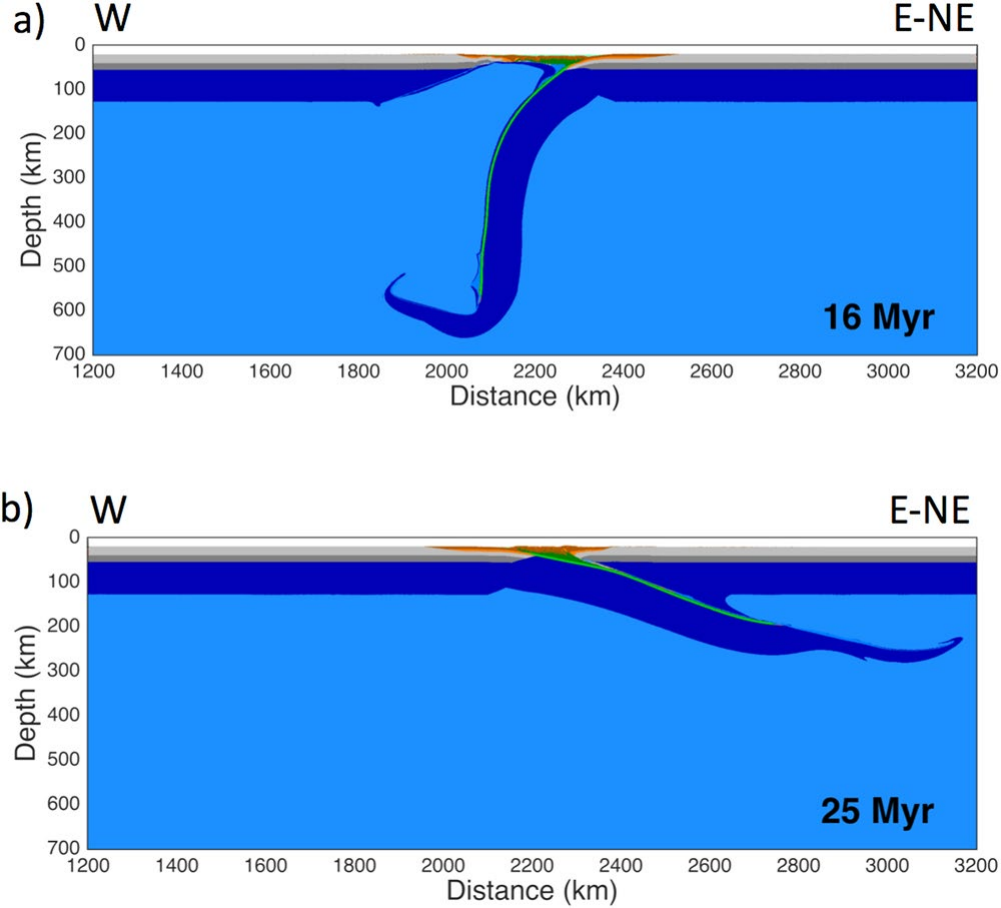
Panel (**a**) shows a W-directed slab. All numerical models present pre-defined rightward subduction polarity; therefore this model was mirrored for better comparison with nature. In panel (**b**) a slab along E or NE-subduction zone is designed. In each model a horizontal mantle flow is imposed, having concordant or opposite direction with respect to the subduction polarity. The difference in dip of the slab is striking: the “westerly” dipping slab is steeper and deeper, whereas “easterly or northeasterly” dipping slab is shallower and less steep. The difference is also remarkable comparing back-arc spreading or not: in fact this latter only occurs in “westwardly” directed slab model (**a**).

Results from a model simulating an “eastward” mantle flow imposed against a “westward” directed subduction (Fig. 2a) show a sub-vertical slab. Subduction initiates via slip along the interplate weak zone (see Supplementary Fig. S3 for further details of the model setup). As some of the initial plate boundary interface material gets subducted, it is replenished by material from the upper layer of the subducting plate, and the hinge begins to retreat. Before the slab tip flattens in the transition zone, the slab is pushed backwards and downwards by the flow. The hinge continues to retreat, allowing the spontaneous formation of back-arc extension at a distinct location with respect to the trench, and causing an uplift of the asthenospheric mantle. The subduction hinge moves away from the upper plate and subduction rates are faster than the convergence rates^12^, leading to a faster recycling of the lithosphere into the mantle^41^. Hinge retreat correlates with the intensity of the mantle wind, regardless of subducting plate strength or age-dependent density. Age variations of the subducting oceanic plate have indeed negligible effects on subduction dynamics (Supplementary Fig. S4 and Supplementary Table S2 – model 7), thus suggesting that mantle wind intensity is a more critical subduction parameter compared to the slab age.

During incipient collision, a large volume of weak crustal material is interposed between the plates. The negative pressure gradient caused by mantle flow and dense retreating slab favor sucking of the mantle into the accretionary prism. As collision proceeds, the rheologically weak part of the crust is scraped off from the retreating lithosphere by the mantle wedge above the subducting slab that acts as a backstop moving in the opposite direction to convergence.

### Models with concordant mantle flow and subduction polarity

Numerical results from the model simulating an “eastward” mantle flow interacting with an “eastward” directed subduction are strikingly different (Fig. 2b); the “eastward” mantle flow holds the subducting slab up, thus resulting in a less steep dip angle and a shallower depth of the slab itself. In this case the subduction hinge is moving toward the upper plate, which is set under compression, and the subduction rates are slower than the convergence rates^12^. The strong correlation between topography and slab dip angle with corresponding variations in mantle flow direction suggests a strong relationship with the underlying subduction dynamics. This should be the reason why this kind of subductions have the highest mountain ranges (i.e., the Andes, the Cascade Range, the Alps etc.). Notably, the shallow subduction also produces slower recycling of the oceanic lithosphere into the mantle^41^. As in the previous model, the age of the subducting plate has subordinate effects on subduction dynamics: low dip angle can be reached either by younger and older oceanic plates, despite having different values of Activation Volume and isobaric Thermal Expansion (Supplementary Table S2). The persistence of a mantle flow is therefore crucial to determine the dip angle of the slab and the state of stress within the upper plate.

At the onset of collision, the buoyancy of the continental crust slows down the convergence rate. As collision continues, crustal material is accreted at the margin, raising the topography and thus building up the compressional stresses within the upper plate. The final structure is that of a narrow and thick collisional zone delimitated by a shallow-dipping slab and characterized by diffuse deformation.

These results thus suggest that when these heterogeneities (that are, concordant mantle wind and a weak asthenospheric layer) are combined in a single model, the dynamics of the subducting plate and the topography evolution of the overriding plate can all be reconciled. Results demonstrate that symmetric changes in the mantle wind direction have an impact on the total force propagated to the upper plate and influences the plate motions, dips and vertical stresses, reflected as a topographic high in the overriding plate.

## Discussion

**Figure 3:**
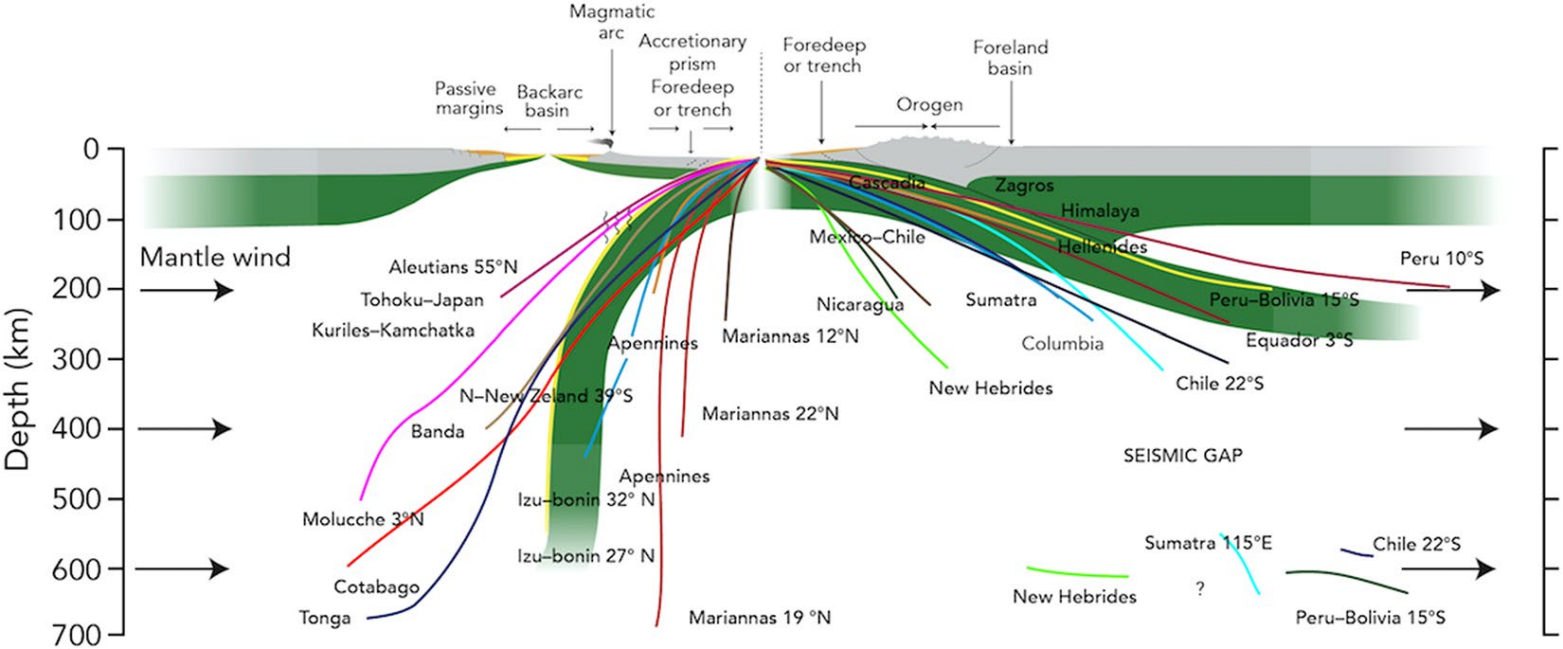
This picture shows our two models (in green), compared with a compilation of the slab dip measured along cross-sections perpendicular to the trench of most subduction zones. Each line represents the mean trace of the seismicity along every subduction. Some E- or NE-subduction zones present a deeper scattered cluster of hypocentres between 550–670 km. Dominant down-dip compression occurs in the W-directed intraslab seismicity, whereas down-dip extension prevails along the opposed E- or NE-directed slabs. The W-directed slabs are, on average, dipping 65.6°, whereas the average dip of the E- or NE-directed slabs, to the right, is 27.1° (modified)^11^. In our models the dip of the slab fits within this average by assuming intensity of the horizontal mantle flow of 3 cm yr^−1^. In this figure the differences in topography and state of stress between the upper plates of both models can be seen.

Our numerical models suggest that the dip of the slab consistently changes as a function of intensity of the mantle wind, whereas the presence or absence of the LVZ is demonstrated to only play a collateral role (Supplementary Figs S6 and S7). This is confirmed by the fact that in our models, negative buoyancy does not influence the slab dip angle and the density contrast between the lithosphere and the hosting mantle is about 35–40 kg/m^3^ on average (for example, in Fig. 2a)^38, 39^. The resulting dip angle associated to a horizontal mantle flow mimics the natural data, providing a different clue to explain the global asymmetry of slab dip. Comparing our models with a global compilation of slab dips (Fig. 3) measured along cross-sections perpendicular to respective trenches^11^, a good fit can be obtained, assuming mantle flow intensity of 3 cm yr^−1^. Dip angle of our “westward” directed slab lies indeed within the average of the W-directed subduction zones worldwide (being ~73.7° the average dip angle for our “westward” directed slab, Fig. 3). Following the same line of reasoning, our “eastward” directed slab (being its dip angle ~17.5°, Fig. 3) correlates remarkably with the average dip of slabs along E to NE-subduction zones. The catalogue in Fig. 3 includes a number of sections for western and eastern sides of the Pacific Ocean, ranging from 55N to 40S latitude degrees and from 50N to 22S latitude degrees, respectively. These can be used as representative for W- and E- to NE-directed subduction zones spread out in opposite Pacific Ocean sides. Also, looking at tomography^42, 43^ (considering the segment of the slab on which seismicity is plotted) and other seismicity data^44–46^, a quite good correlation between slab dip and subduction polarity can be observed. Exceptions are for Northeastern-Japan, Java and Central America, these latter two being more likely related to the obliquity of the slab direction with respect to the main convergence direction. The northern-Japan setting is peculiar because the subduction hinge is now converging relative to the upper Eurasian plate, hence inverting the previous slab retreat relative to the upper plate and the contemporaneous opening of the Japan Sea^12^.

Although a possible link between slab dip, deformation of the overriding plate and trench motion has been already proposed^2, 5^, our results could be useful to explain some of the different features between Andean- and Mariana-type subductions in a different way. In the past^2^, differences between these two subduction types were explained by ablation extent during subduction process. In our conceptual and numerical models, the absence of crustal shortening in Mariana-type subductions should be due to the back-arc basin opening, as a consequence of the slab rollback associated to the retreat of the subduction hinge relative to the upper plate, and shallow décollement levels determine mostly thin skinned tectonics. In such settings most of the crust and the lithospheric mantle are completely subducted instead of being involved in the accretionary prism building. In the opposite subduction setting, the slab hinge converges relative to the upper plate and the deep décollement levels allow involvement of rocks coming from a higher depth in orogens building. Some authors^5, 47^, then, used seismicity data coming from deeper and shallower parts of the slab, separately, to study relations between slab dip and subduction direction. However, it is important to notice that splitting a slab in a shallow and a deep part of it could be misleading: (1) in subduction zones where there is a continental upper crust (approximately all E- to NE-directed), the behaviour of the first 125 km is mostly influenced by the thickness of the lithosphere and by the slab angle with respect to convergence direction. Slab dip might not even show such a different dip in its shallow part because of the presence of the décollement level (LVZ, considered in our numerical models), located between a 100 and 200 km depth. However, regional monocline dip reveal the same asymmetry between W-directed an E- to NE-directed subduction zones^17^. (2) Seismicity below W-directed and E- to NE-directed subduction zones is quite different: E- to NE-directed subduction zones have a seismic gap between 250–300 km and a 500 km depth, and seismic isolated events at deeper depth origin are still unclear^7^.

Another issue that should be considered when evaluating relationship between subduction zones direction and slab dip, is that most of subductions worldwide show an arcuate geometry^7, 48^; therefore slabs could form different angles with subduction direction and, consequently, they have different dip angles according to their obliquity with respect to the main convergence direction. Furthermore, some works^5^ consider trench-perpendicular migration velocities but, doing this, an assumption is made: in fact, when having an oblique trench with respect to subduction direction the hinge moves obliquely too, due to stress deviation from the convergence direction and strain partitioning. Moreover, hinge velocity cannot be measured in a precise way, especially when calculated with respect to the mantle^7^.

However, here we analyse the possibility that a global feature –such as the mantle wind– could be the first-order controlling parameter of slab dip and stress regime within the upper plate^7^. Our results show a back-arc basin opening only in models with “westward” directed slabs, as it can be seen along W-directed subduction zones worldwide (i.e., Appenines, Marianas, Tonga-Kermadec, Sandwich etc.). This opening could thus be critically related to the slab rollback, due to the push exerted from the “eastward” mantle flow on a “westward” directed subducting slab. In fact, it has to be considered that extension within the overriding plate, in the two subduction end-members, has different geologic origins: along W-directed subduction zones, back-arc spreading occurs as a consequence of slab rollback and of the asthenospheric replacement for the retreated lithosphere (as it can be seen also from our W-directed subduction model), whereas for E-NE-directed subductions back-arc basins open in few places where the upper plate lithosphere is split into two sub-plates that have different velocity with respect to the same lower plate^12^. Moreover, the first one are characterized by fast back-arc opening, widely distributed throughout the upper plate and eventually arriving to oceanization (e.g., the Western Mediterranean) whereas, in the second one, extension within the upper plate is confined in areas close to the transfer zones (e.g., the Aegean Sea) and rarely reaching the oceanization stage (e.g., the Andaman Sea).

Our experiments suggest, thus, that the existence of a predominantly “eastward” horizontal mantle flow (along the so-called, undulated, tectonic equator) may explain several contrasting characteristics of subduction zones worldwide. The subduction of the Nazca plate beneath the Andes provides a key test for this analysis. In fact, in the kinematic model of a slow net rotation of the lithosphere driven only by the slab negative buoyancy, beneath the Nazca plate and the slab of the south America cordillera, the mantle flow should be westerly directed, providing a steep slab. However, as shown in the numerical modelling presented here, the shallow dip of the Andean slab is consistent with an eastward mantle flow even beneath the Nazca plate as evident by shear wave splitting results^49^ and providing indirect support for the fast lithospheric rotation rates relative to the mantle^32^.

The origin of the hypothesized global mantle wind is largely enigmatic and goes beyond the scope of our study. While geophysical constraints support the hypothesis of an E-directed mantle flow over at least the last 100 Myr^15^, how mantle structure and kinematics may have behaved in the geological past is still unclear. One possible explanation is that the lithosphere is sheared “westward” relative to the asthenospheric mantle, along the mainstream of plate motion in which W-directed subduction zones contribute to a three times larger fraction of global lithospheric recycling, compared to E- and NE-directed subduction zones^41^. As a result, a larger fraction of the asthenospheric mantle material has to move to the east, thus creating a global mantle wind. However, understanding of physical origin and distribution of the horizontal mantle flow requires self-consistent global mantle convection and plate tectonics modelling^36^ and remains as a challenge for future research.

## Methods

The numerical experiments were carried out with the code I2VIS^40^. This code is based on a combination of the finite difference method applied on a staggered Eulerian grid with a marker-in-cell technique. The initial set-up is shown in Supplementary Fig. S3. The momentum, continuity and energy equations are solved in the Eulerian frame, and physical properties are transported by Lagrangian markers that move according to the velocity field interpolated from the fixed grid. We use non-Newtonian viscous-plastic rheologies (Supplementary Table S1) in a model that is fully thermodynamically coupled and accounts for mineralogical phase changes^50^, as well as for adiabatic, radiogenic and frictional internal heating sources. The viscous-ductile rheological term accounts for power-law and diffusion creep as well as for Peierls creep at depth. The experiments were performed in a 4000 × 1400 km computational domain. The models used a grid resolution of 1361 × 351 nodes with variable grid spacing. This allowed a high grid resolution of 1 km in the subduction area subject to largest deformation. More than 12 million Lagrangian markers carrying material properties were used in each experiment. The free surface upper boundary is simulated using the “sticky air” technique^51^, enhanced by the high-density marker distribution in the near-surface. Two 1700-km-long continental plates were separated by a 700-km-long oceanic plate. Both continental plates and the oceanic one are composed of an upper crust, lower crust and lithospheric mantle. For all models presented in this work, periodic boundary conditions have been implemented on the left and right boundaries following the same approach as in previous numerical experiments^52^, and free slip condition is applied at the top and the bottom of the computational domain. A low-viscosity-zone between a 100 and 200 km depth, and an imposed throughgoing asthenospheric mantle flow of −3 to +3 cm yr^−1^ are implemented in the models. The viscosity of this weak layer and the mantle flow velocities applied are listed in Supplementary Information and Supplementary Table S1, respectively. Full details on the method, allowing its reproduction, are provided in Supplementary Information and in ref. 40. This algorithm has been thoroughly tested in two dimensions, and used for lithospheric deformation experiments in a number of previous studies. All data used in this work can be accessed from the sources provided in the reference list and the Supplementary Information.

## Electronic supplementary material

Supplementary Information - Horizontal mantle flow controls subduction dynamics
