# Supplementary material for: Horizontal mantle flow controls subduction dynamics
Source: Sci Rep. 2017 Aug 8;7:7550. doi: 10.1038/s41598-017-06551-y (PMC5548891; doi:10.1038/s41598-017-06551-y)
Supplement: Supplementary file 1 — Supplementary Information - Horizontal mantle flow controls subduction dynamics [file 41598_2017_6551_MOESM1_ESM.pdf]

Supplementary Information

Horizontal mantle flow controls subduction dynamics

E. Ficini[1,*], L. Dal Zilio[2], C. Doglioni[1,3], T. V. Gerya[2]

[1] *Department of Earth Sciences, Sapienza University of Rome, Rome, Italy*
[2] *Institute of Geophysics, ETH Zurich, Zurich, Switzerland*
[3] *Istituto Nazionale di Geofisica e Vulcanologia, INGV, Rome, Italy*
** Correspondence and requests should be addressed to E. Ficini (eleonora.ficini@uniroma1.it)*

1. General concept

Our models aim to improve the physical understanding of plate tectonic dynamics, with particular emphasis on the role of a horizontal mantle flow on subduction dynamics and resulting topographic signature. Geophysical data support the hypothesis of a preferential "easterly" directed flow of the Earth's mantle. This flow is undulated between the Western Pacific and the Eastern Africa, and it seems to be valid at least for the last 40 million years[21,32]. This mantle flow, of yet enigmatic origin, would be the first order parameter influencing both subduction dynamics and the relative orogens (Supplementary Fig. S1). In fact, there are striking differences if looking at subduction zones following or not this counterflow.

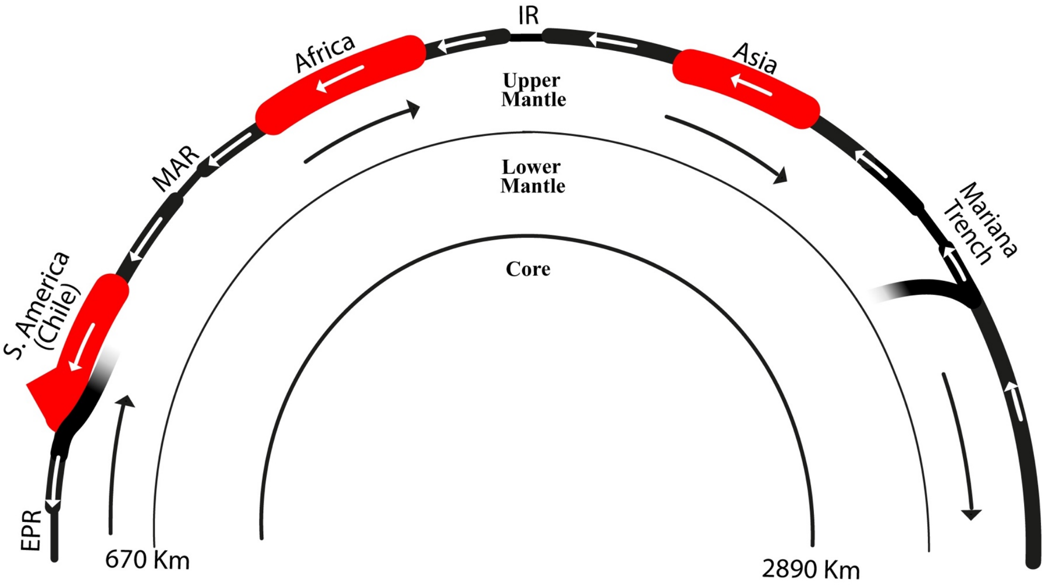

Supplementary Fig. S1 – Cartoon of the lithosphere motion (white arrows), along the Tectonic Equator, with respect to the opposite motion of the mantle (black arrows). Subduction zones along South America are characterized by shallow and less steep slabs with high topography orogens, while in subduction zones like the Marianas, the slab is steeper, and present a characteristic back-arc basin in the upper plate.

In our manuscript we mentioned the evolution of the topography and the development of a back-arc basin in the two end-member cases (Supplementary Fig. S2).

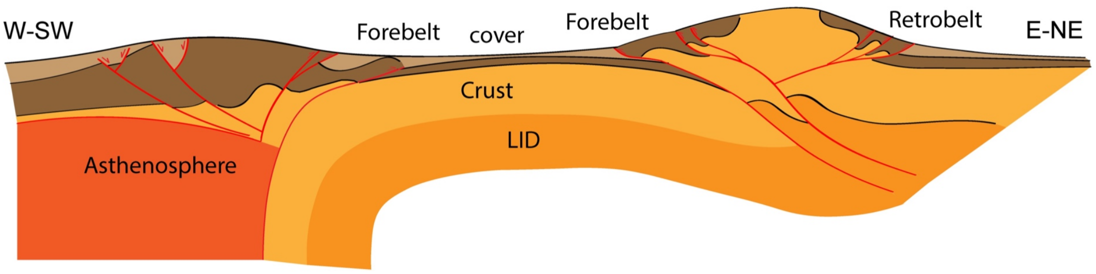

Supplementary Fig. S2 – In this figure the two topographic end-members can be seen. To the right the E- or NE-directed slab is shown: a high topography double-verging orogen forms during the evolution of the subduction (e.g., Andes, Alps, Himalayas). To the left the opposite subduction setting forms an orogen with a lower topography, single-verging shallow accretionary prism and a shallow asthenospheric mantle wedge (e.g., Marianas, Barbados, Apennines).

The W-directed subduction zones show steep slabs, single vergent orogens with low topography and a well-developed foredeep. This kind of orogens involve young and shallow rocks coming from superficial offscraping of the plates involved in the subduction process. In this case the basal décollement of the subducting plate is never connected to the surface but is rather folded and swallowed down inside the subduction zone (e.g., Marianas, Tonga-Kermadec and Barbados), being thus unable to feed the accretionary prism with rocks coming from high depths. On the other hand, E- to NE-directed subduction zones show shallower slabs, highly-coupled collision zones with double vergent orogens, two shallow slow-subsiding foredeeps[53,54], and high topography involving older and deeper rocks because of the deeper décollement planes (e.g., American cordilleras, Alps, Zagros, Himalayas), being thus able to involve the basement of the subducted plate during the collisional stage.

2. Modelling approach: General features

For this study, we used a thermo-mechanical 2D code I2VIS[40,55] based on finite differences (FD) method and marker-in-cell (MIC) technique. The conservative finite-difference schemes are designed over a non-uniformly spaced fully staggered Eulerian grid. The material properties are advected by Lagrangian markers according to a computed velocity field using a fourth-order in space, first order in time explicit Runge-Kutta scheme. The material properties are interpolated from the markers to the

grid by using a distance-dependent averaging. Full details of this method, allowing for its reproduction, are provided elsewhere[40,55].

2.1. Governing equations

The I2VIS code solves for the conservation of mass, momentum and energy. Conservation of mass is approximated by the incompressible continuity equation:

$$\frac{\partial v_x}{\partial x} + \frac{\partial v_y}{\partial y} = 0; \qquad (1)$$

where x and y denote horizontal and vertical Cartesian coordinates. The momentum equations are presented in the form of the Stokes flow approximation:

$$\frac{\partial \sigma'_{ij}}{\partial x_j} - \frac{\partial P}{\partial x_i} + \rho g_i = 0 \qquad (2)$$

which in 2D reads:

$$\frac{\partial \sigma'_{xx}}{\partial x} + \frac{\partial \sigma'_{xy}}{\partial y} - \frac{\partial P}{\partial x} = 0 \qquad (3.1)$$

$$\frac{\partial \sigma'_{yy}}{\partial y} + \frac{\partial \sigma'_{yx}}{\partial x} - \frac{\partial P}{\partial y} = -\rho g_y \qquad (3.2)$$

where σ'_{ij} are the components of the viscous deviatoric stress tensor, ρ is the density dependent on rock composition, temperature and pressure, and g_y is the acceleration due to gravity. The components of the deviatoric stress tensor (σ'_{ij}) are calculated using the incompressible viscous constitutive relationship between stress and strain rate ($\dot{\varepsilon}$), as follows:

$$\sigma'_{ij} = 2\eta\dot{\varepsilon}_{ij} \; ; \quad (4)$$

$$\dot{\varepsilon}_{ij} = \frac{1}{2}\left(\frac{dv_i}{dx_j} + \frac{dv_j}{dx_i}\right); \qquad (5)$$

The conservation of energy equation describes the temperature changes in a continuum due to internal heat generation/consumption and advective/conductive heat transport:

$$\rho c_{P\_eff}\left(\frac{\partial T}{\partial t} + v\, grad(T)\right) = -\frac{\partial q_x}{\partial x} - \frac{\partial q_y}{\partial y} + H_r + H_a + H_s, \qquad (6)$$

where q_x and q_y are heat flux components; $k = f(P, T, C)$ is thermal conductivity

which depends on the pressure-temperature conditions and composition. H_r, H_a and H_s are, respectively, radioactive, adiabatic and shear heat production. Radioactive heat production depends on the rock type and it is assumed to be constant through time. The adiabatic heat production/consumption is related to pressure changes (compression-decompression):

$$H_a = T\alpha_{eff}\left(v_x\frac{\partial P}{\partial x} + v_y\frac{\partial P}{\partial y}\right). \quad (7)$$

The shear heat production is given by the dissipation of mechanical energy during viscous deformation and depends on the deviatoric stress and deviatoric strain rate:

$$H_s = \sigma'_{xx}\dot{\varepsilon}_{xx} + \sigma'_{yy}\dot{\varepsilon}_{yy} + 2\cdot\sigma'_{xy}\dot{\varepsilon}_{xy}. \quad (8)$$

The effect of latent heating related to the phase transformations of the rocks is included implicitly by calculating the effective heat capacity (c_{P_eff}) and the effective thermal expansion (α_{eff}) through thermodynamic relations[55]. Phase transitions are parameterized as a function of thermodynamic state variables (P, T, V) and composition by using polynomials to interpolate the reaction boundary[56]. For instance, the olivine polymorphic transformations and the post-spinel reaction are parameterized with the linear *P–T* relationship:

$$P = P_0\frac{\partial P}{\partial T}\Delta T = P_0 + \gamma\Delta T\ ; \quad (9)$$

where γ is the Clapeyron slope of the reaction, P_0 is the pressure at reference temperature (T_0), and $\Delta T = T - T_0$.

The visco-plastic behaviour is implemented via evaluation of the effective viscosity of the material. The viscous properties are computed by taking into account the contribution of different creep mechanisms such as diffusion, dislocation and Peierls creep, as:

$$\frac{1}{\eta_{eff}} = \frac{1}{\eta_{diff}} + \frac{1}{\eta_{disl}} + \frac{1}{\eta_{peierls}}, \quad (10)$$

where η_{diff}, η_{disl} and $\eta_{peierls}$ are calculated from flow laws[37]. The strength of the material is limited by:

$$\eta_{eff} = \frac{\sigma_{yield}}{2\dot{\varepsilon}_{II}}\,, \quad (11)$$

where the yield stress is described at shallow depths by the Drucker-Prager yield criterion:

$$\sigma_{yield} = cos\phi \cdot C + \ sin\phi \cdot \ P\left(1 - \lambda_{fluid}\right). \quad (12)$$

Here, ϕ is the internal friction angle, C is the cohesion, and λ_{fluid} is the assumed pore fluid pressure factor.

2.2. Model setup

The initial setup for our models is shown in Supplementary Fig. S3, while rheological[37] and thermal[57] parameters of materials used for the experiments are shown in Extended Table S1. The computational domain size is 4000 x 1400 km and is discretized with 1361 x 351 Eulerian nodes, with more than 11 million randomly distributed Lagrangian particles. This allowed a minimum grid resolution of 1 km in the area subject to largest deformation. Elsewhere, the resolution increases gradually from 1 km up to 5 km. Vertical resolution is 1 km for the first 100 km and then increases with depth to a maximum of 5 km. Two 1700-km-long continental plates were separated by a 700-km-long oceanic plate. Both continental plates and an oceanic are composed of an upper crust, lower crust and lithospheric mantle. The initial continental geotherm was set using values of 273 K at the top and 1617 K at the bottom of the lithosphere (100-km-thick). The thermal structure of the initial plate set-up is computed according to the half-space cooling model[58] for a given age between 20 and 150 Ma. The thermal gradient used within the mantle was quasi-adiabatic (0.5 K/km). To ensure an efficient heat transfer from the surface of the crust, the temperature of the "sticky air" is kept constant at 273 K. Gravitational acceleration of g_y = 9.81 m/s was used in the model. Subduction starts as the result of the imposed rightward convergence rate of 5 cm yr^{-1} imposed for the first 6 Ma on the left plate. The subduction is localised along a prescribed inclined rheologically weak (hydrated) intra-plate zone characterized by low plastic strength of 1 MPa[56] (Supplementary Fig. S1). The subducting plate is also decoupled from the left model boundary by a low-viscosity zone. We also impose low plastic strength for the fluid-saturated subducting basaltic crust (see Supplementary

Table S1) acting as a lubricating layer[59,60]. This ensures that the induced one-sided subduction can continue spontaneously after 6 Myr period of initial plate convergence. A low-viscosity layer (10^{18} Pa s) between 100-200 km depth has been included in some numerical experiments (see Supplementary Table S2) in order to test the role of a weak and partially melted asthenosphere, as suggested in Ref. 61. Finally, as the main additional ingredient of our models, we impose a throughgoing horizontal asthenospheric mantle flow (see Boundary Conditions section).

During numerical experiments, a suite of parameters has been tested to investigate how they affect the dynamic of the model itself. In particular, we considered the activation volume, the coefficient of thermal expansion, the age of the subducting lithosphere, the direction and velocity of the horizontal mantle flow (Supplementary Tables S1 and S2). We chose to observe the behaviour of the models changing these parameters to see how variations in effective viscosity and in density, with temperature and depth, influence subduction dynamics (Supplementary Table S2), in the set of conditions of a mantle flow acting as a push or as a sustain for a subducting slab, including the effect of a décollement level. Furthermore, also the age of the subducting oceanic plate was taken into account: in fact, we wanted to numerically and physically demonstrate that the horizontal mantle flow has a sufficient strength to generate a steep dip angle also in slabs that are made of young (and therefore hot) oceanic lithosphere. This latter represents a further evidence that the dynamics of a subducting slab and, more generally, of plate tectonics is far from being completely known, not being the slab pull force (if any) alone a full explanation for these mechanisms.

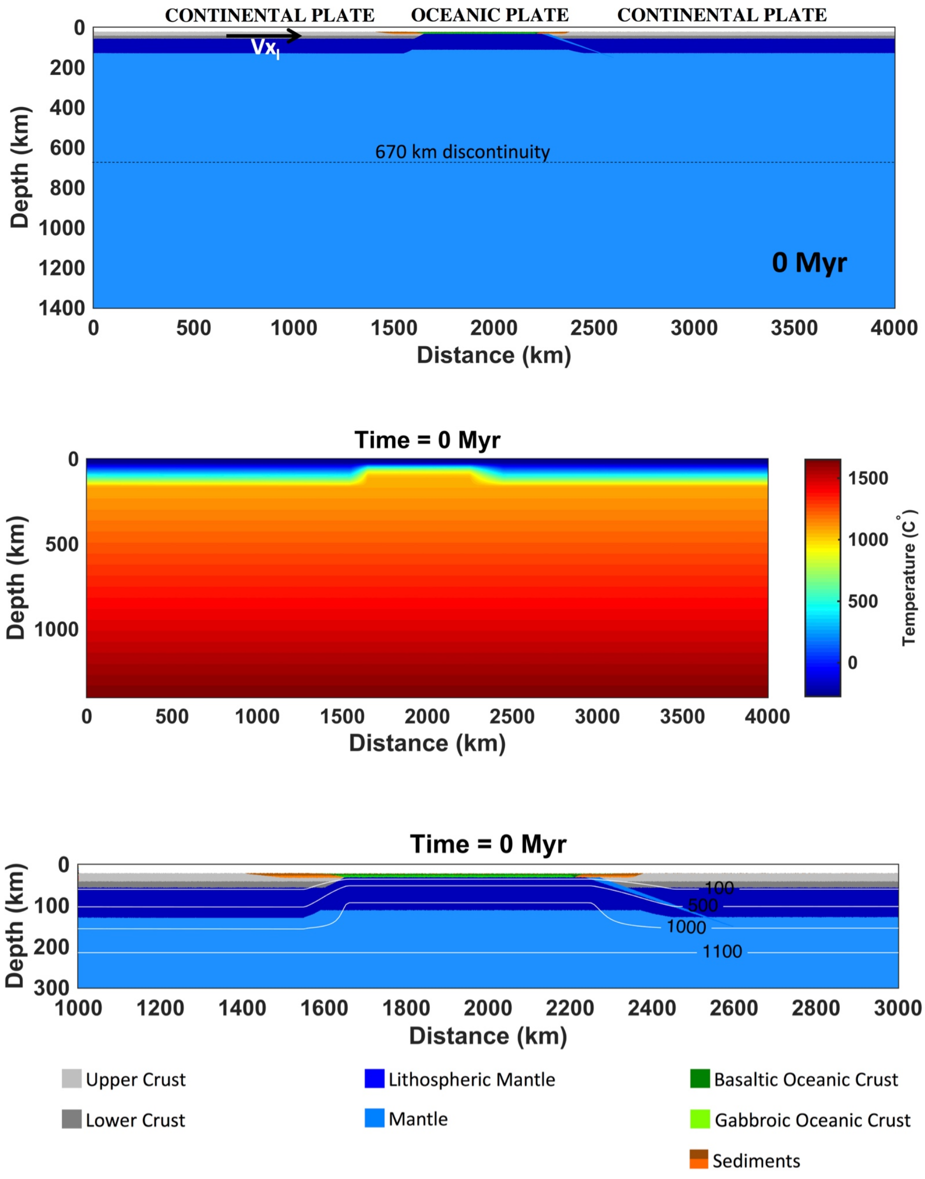

Supplementary Fig. S3 – Model setup. In the bottom panel isotherms are the white lines. The light blue line in the lower panel (between the oceanic, lower plate and the upper continental right plate) is the weak zone. Plate convergence rate of 5 cm yr^{-1} is applied at 1000 km for 6 Myr to initialize the subduction process. Color code for different materials is shown at the bottom of the figure.

2.3. Boundary conditions

For all models presented in this work, the boundary conditions are set to be periodic on the left and right boundaries, and free slip condition is applied at the top and the bottom of the computational domain. A free slip condition requires that the normal velocity component on the boundary is set to zero whereas the tangential component do not change across the boundary (this condition also implies zero shear strain rates and stresses along the boundary). The free slip condition at the top and bottom boundaries is then defined as follows:

$v_y = 0;$ (13)

$\frac{\partial vx}{dy} = 0$; (14)

Additionally, a 10–km–thick layer of "sticky air" (ρ_{air} = 1 kg/m^3, η_{air} = 10^{18} Pa s) was utilized in order to mimic the effect of a free surface and the development of topography[62]. In order to simulate a polarized troughgoing asthenospheric mantle flow, periodic boundary conditions are established on the left and right boundaries by discretizing the fundamental equations across the boundaries and by prescribing the horizontal velocity (v_x) and the temperature (T) unknowns on both sides of each boundary as identical. From a physical point of view, this implies that these two boundaries are open and that flow leaving the model through one boundary immediately re-enters through the opposite side. This condition is often used in mantle convection modelling to simulate part of a cylindrical shell domain, or mimic it, in Cartesian coordinates[63], and is formulated as follows:

$v_{x(N-1)} = v_{x(1)}$; (15)

$T_{(N-1)} = T_{(1)}$; (16)

where N is the number of nodes. Periodic boundary condition at the right boundary was combined with prescribed horizontal velocity at the left boundary: zero horizontal velocity was prescribed for the lithosphere, whereas constant inflow/outflow horizontal velocity condition was applied for the entire asthenosphere (Supplementary Fig. S4). To evaluate the role of mantle flow velocity we conducted numerical experiments with mantle wind rates covering the full spectrum from -3 to +3 cm yr^{-1} (Table S2)

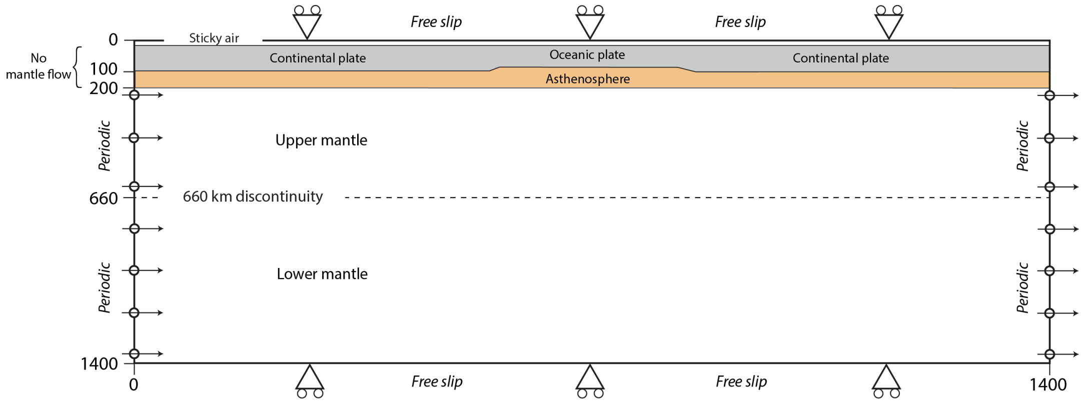

Supplementary Fig. S4 – Schematic model setup used for standard 2D experiments in Cartesian geometry and mechanical boundary conditions.

3. Supplementary results

In the following section, we show some models of W-dipping slabs and E-dipping slab (Supplementary Figs. S5 and S6). In Supplementary Fig. S5 a westward-like subduction model can be seen. This model has an older age of the oceanic subducting plate and a lower velocity of the mantle wind, with respect to the model in Fig. 2a of the paper (Supplementary Table S2, model 7 and a, in bold, respectively). In this case the dip angle of the slab is steep (~45°), but not as much as the one of model in Fig. 2a of the paper, although its older age. This leads to the conclusion that the age of the slab has a minor role in affecting subduction dynamics, with respect to the role of the velocity of the horizontal mantle wind. In fact, model 7 here, has a lower dip angle due to the lower velocity of the mantle flow pushing the slab downward. A back-arc basin is opening (as in all westward-like models cases).

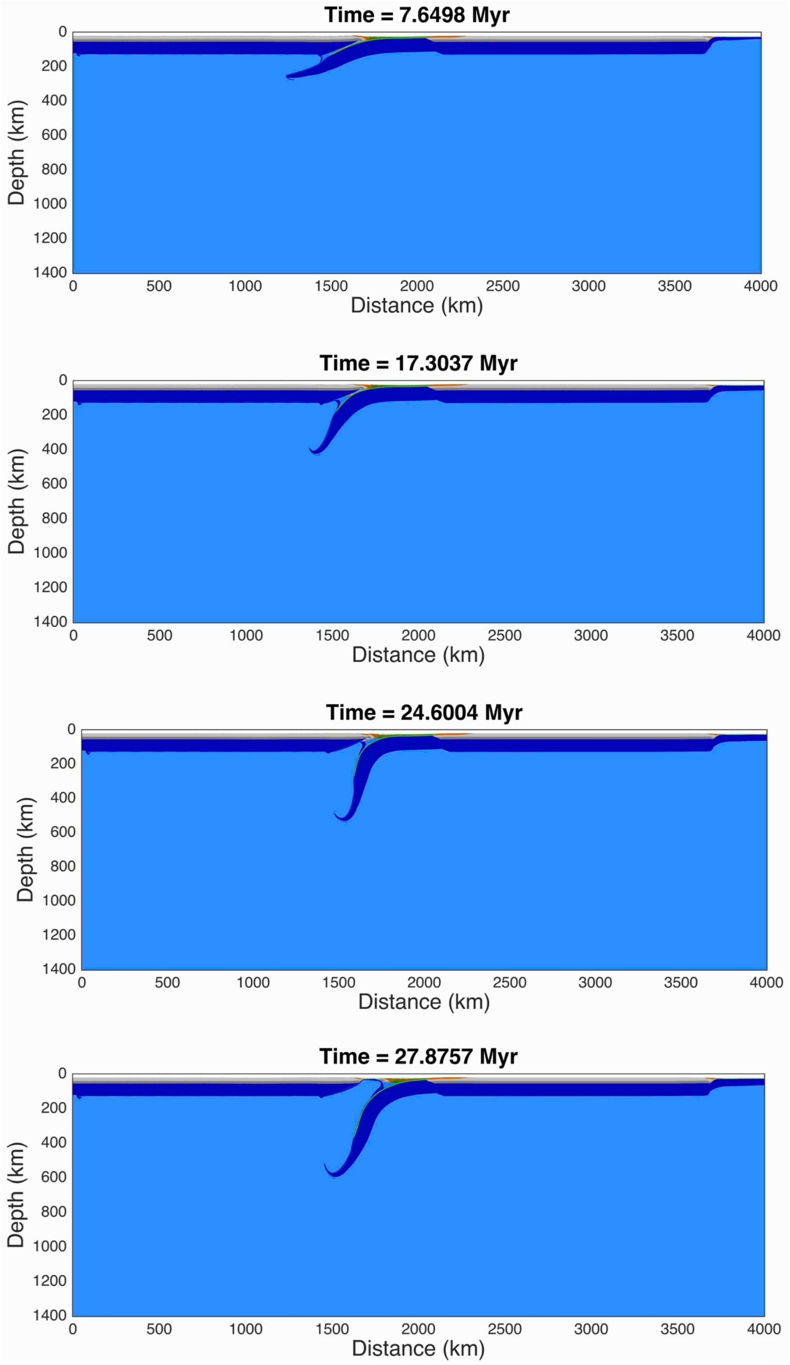

Supplementary Fig. S5 – Westward-like subduction model with LVZ and horizontal eastward mantle wind. All panels have been mirrored for better comparison with nature.

In Supplementary Fig. S6 an eastward-like subduction is shown. The slab has a low dip angle (Supplementary Table S2, 13) and at some point (at around 19.5 Myr) collision occurs and, therefore, there is not back-arc spreading in the upper plate. In this case, the dip angle is higher than in the "eastward-like" paper model (Supplementary Table S2, model b, in bold), probably due to the double effect between a high Thermal Expansion value and a high Activation Volume value. Despite this, we have the same sustaining effect of the mantle acting on the subducting slab that model with the eastward-directed subduction shows in the paper (Fig. 2b).

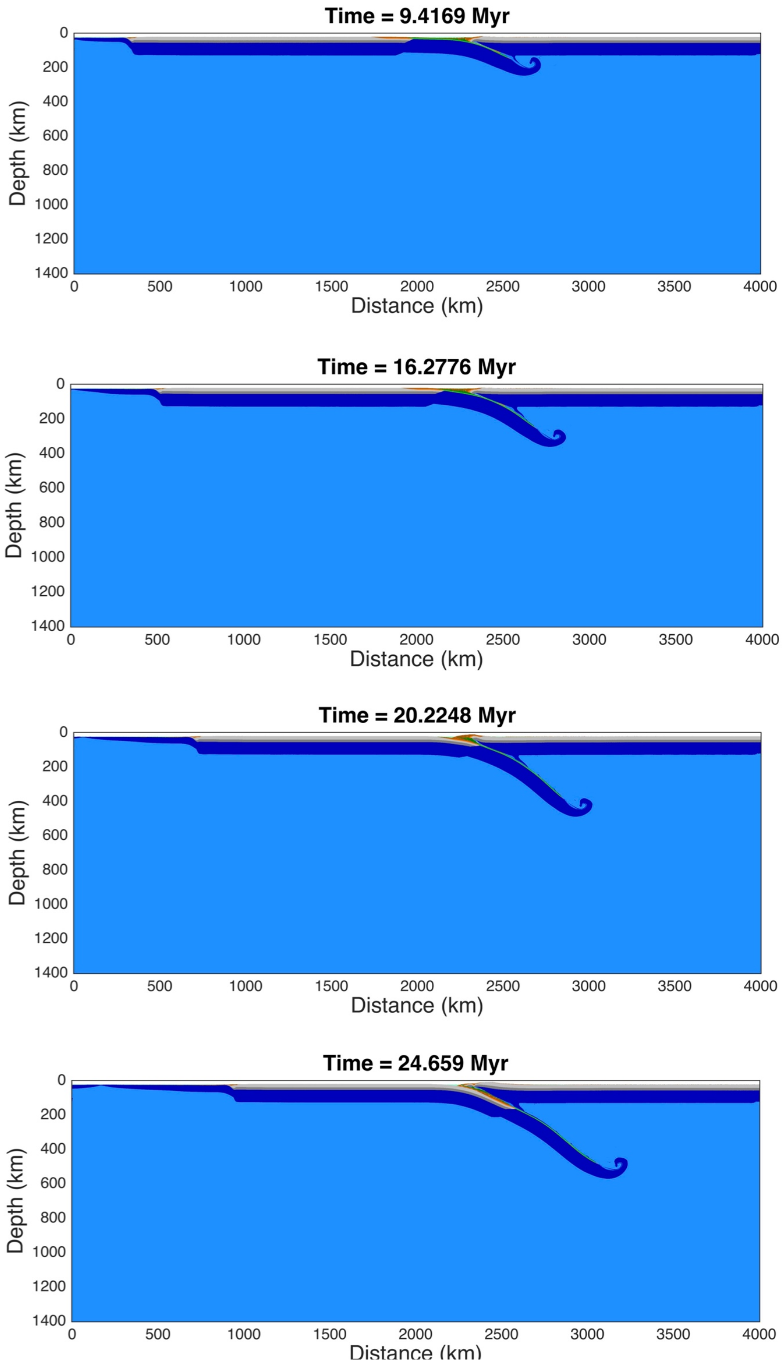

Supplementary Fig. S6 – Eastward-like subduction model with LVZ and horizontal eastward mantle wind.

Moreover, we compared all our models with a number of reference models without LVZ (Supplementary Figs. S7 and S8 and Supplementary Table S2, Ref. 1, 2), without the horizontal mantle flow applied (Supplementary Fig. S9 and Supplementary Table S2, Ref. 3), alternatively, and with a model without both the mantle wind and the LVZ (Supplementary Fig. S10 and Supplementary Table S2, Ref. 4). Starting from models

without LVZ, in Supplementary Fig. S6 the mantle flux is considered as negative (i.e., with an opposite direction with respect to the subduction direction): the dip of the slab has a steep angle (Supplementary Table S2, Ref. 1), and there is a back-arc opening on

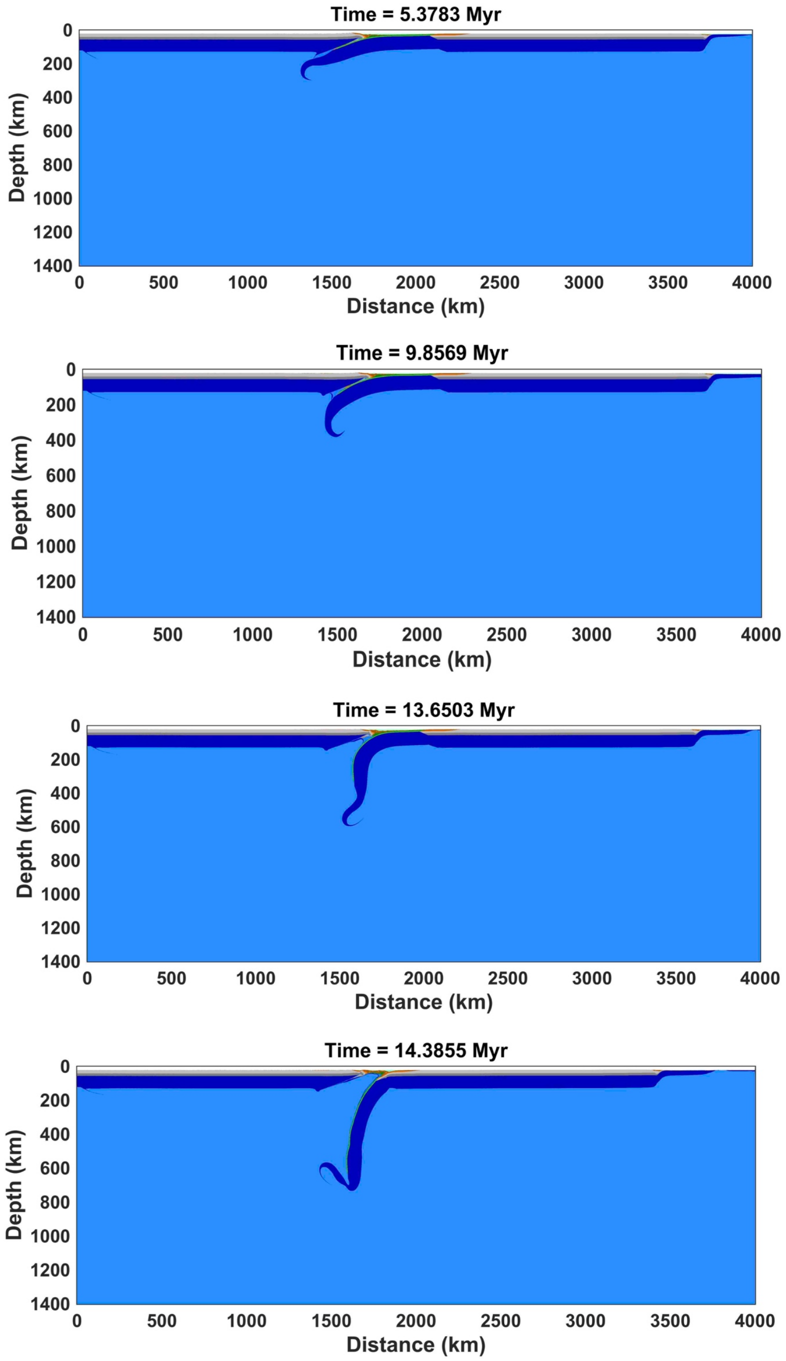

Supplementary Fig. S7 – Westward-like subduction model without LVZ, with the horizontal eastward mantle flow. All panels have been mirrored for better comparison with nature.

the upper plate, like in the other westward-like subduction models (Supplementary Table S2). Subduction in this case is very fast.

In model in Supplementary Fig. S8 (Supplementary Table S2, Ref. 2) mantle flux is considered as positive (i.e., with the same direction of the subducting slab): the oceanic subducting slab is sustained by the mantle flow, but the absence of the decoupling level between the lithosphere and the mantle causes a strong coupling between the upper and the lower plate. In fact, the slab (that has a dip angle of ~17.5°) is right under the upper plate during the whole subduction process (Supplementary Fig. S8).

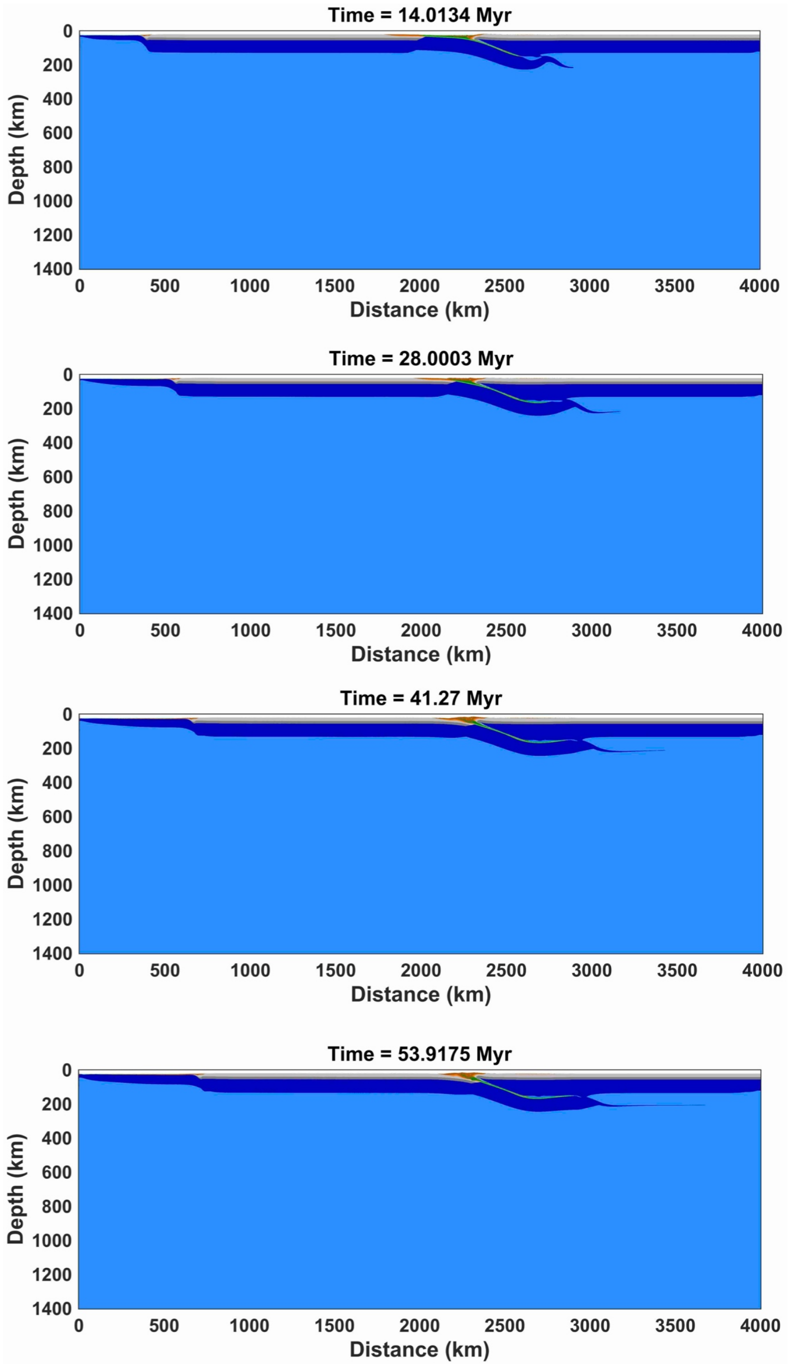

Supplementary Fig. S8 - Eastward-like subduction model without LVZ, with the horizontal eastward mantle flow.

For model without mantle wind (Supplementary Fig. S9 and Supplementary Table S2, Ref. 3), the age of the subducted lithosphere that facilitate subduction is much more higher (150 Myr) than the one used in models with mantle wind. Substantially, slabs undergo subduction for older age values of the oceanic plate. All the slabs presented in this paper, instead, could subduct with younger ages (and this is actually what can be observed in nature). For this model the slab dip angle is about 67° (an almost intermediate value).

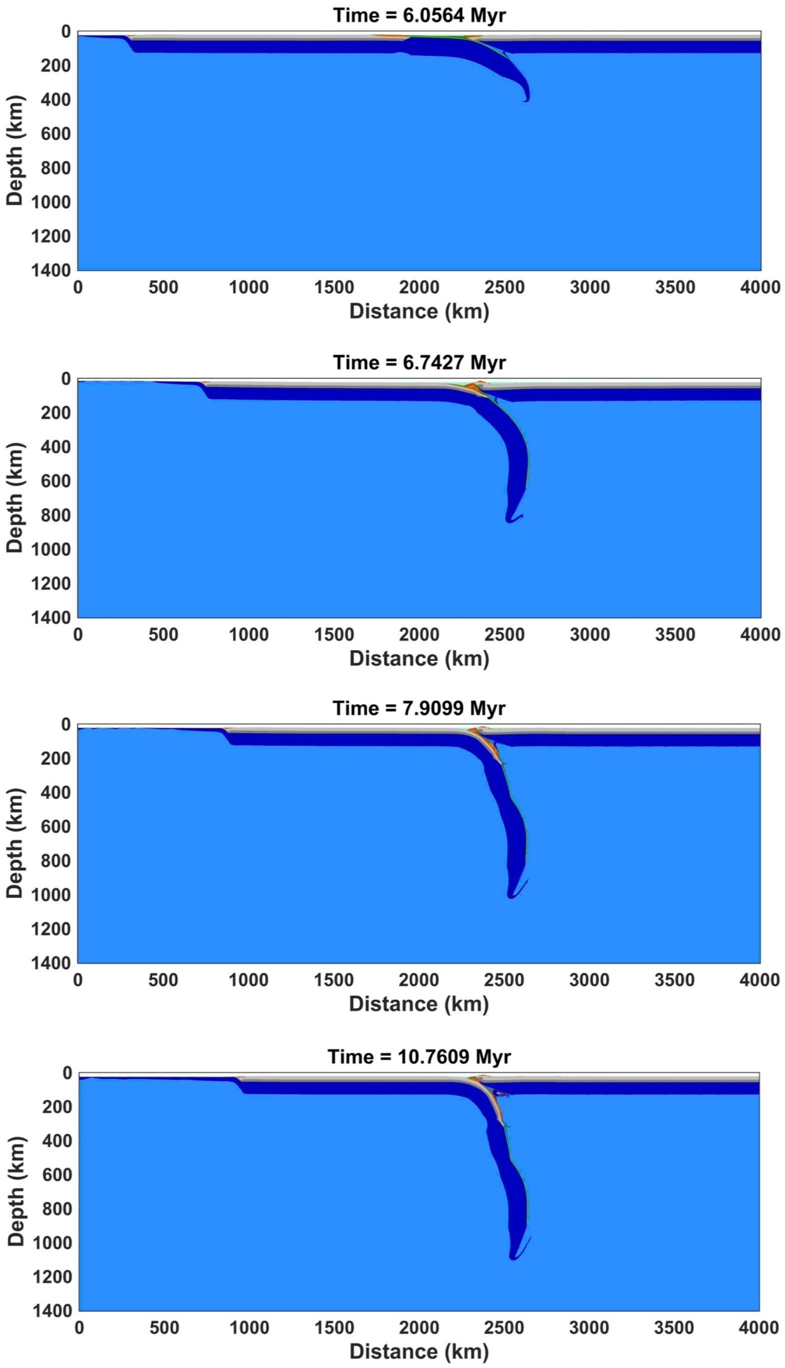

Supplementary Fig. S9 - Subduction model with LVZ, but without the horizontal eastward mantle flow.

Also in case of model without mantle flow and without LVZ (Supplementary Table S2, Ref. 4), the age of the subducted lithosphere required for the sinking of the slab is higher (150 Myr) than the one used for the other models (Supplementary Table S2). In this reference model the dip angle of the subducting slab has an intermediate value (~52.18°, Supplementary Fig. S10) between the E-directed models and the W-directed ones (Supplementary Table S2).

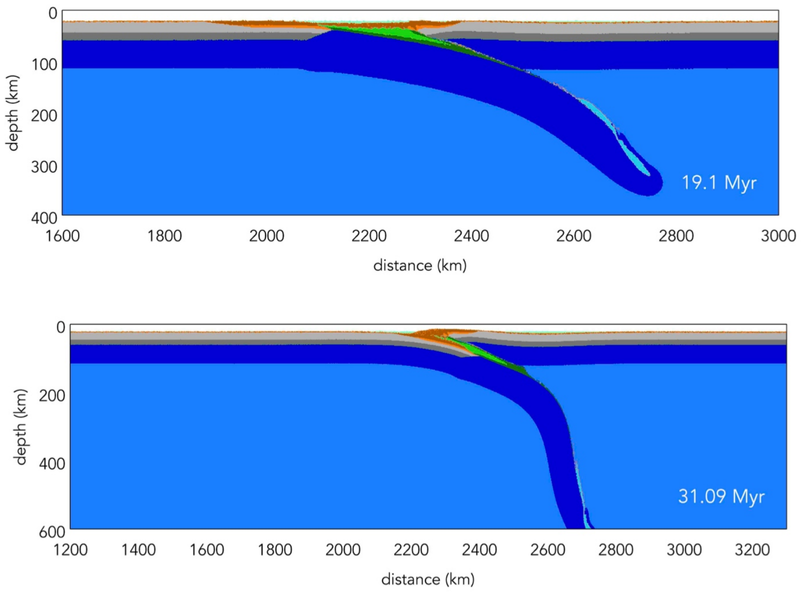

Supplementary Fig. S10 - Subduction model without LVZ and without the horizontal eastward mantle flow.

In conclusion, if we look at Supplementary Table S2 parameters, we can see that all models with negative mantle direction have steep dip angles and back-arc opening on the upper plate. Conversely, all models with positive mantle direction show lower dip angle and no opening of the back-arc basin in the upper plate. Furthermore, for older ages of the oceanic plate, in models with negative direction of the mantle flow, the dip angle of the slab assumes lower values if compared with higher values of the younger aged oceanic plates: this has to be ascribed to the different intensity of the mantle wind pushing on the slab. For older ages of the oceanic plates, in models with positive mantle flow direction, slabs preserve their low dip angle values. Regarding reference models, those without LVZ have a worst dynamic with respect to the ones with LVZ. Nevertheless, the most important observation that we can do, is that in models without mantle flow implemented (Supplementary Table S2, Ref. 3) the slab go through subduction process in an easier way with an older age of the subducting oceanic plate,

while looking at models with mantle wind, the age of the subducting plate would be, thus, of less importance.

The evidence of the two end-members of the subduction setting is clear if comparing the western and the eastern side of the Pacific, however this can be considered as a global trend (Supplementary Fig. S11). In fact, all the plates move toward the west following a mainstream: the so-called Tectonic Equator (Supplementary Fig. S11, yellow line). This should imply a mainly eastward counterflow within the mantle. This counterflow, also as a result of our numerical study, could thus be considered as a first order parameter in controlling subduction dynamics with respect to the geographic polarity of the subducting slab (Supplementary Fig. S11, red and green lines).

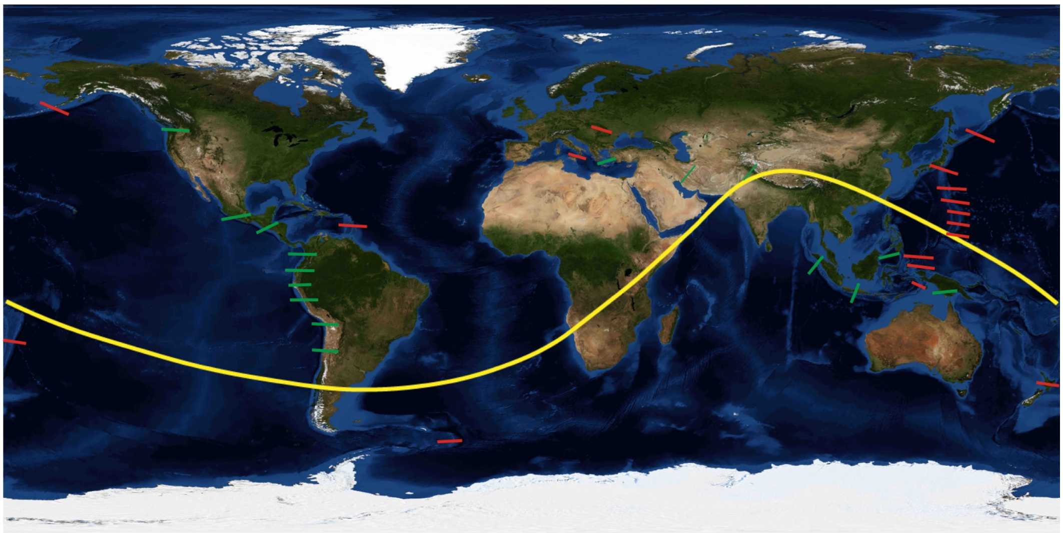

Supplementary Fig. S11 – Image showing the principal subduction zones (red and green lines) worldwide and their polarity with respect to the tectonic equator (yellow line). W-directed subduction zones are the red lines, while E- or NE-directed ones are the green lines. Map generated using GPlates 1.5.0 software – www.gplates.org

Material	k (W/m/K)	H_r (W/m^3)	Cp (J/kg)	sin(ϕ)	C (MPa)	Flow Law	η_0 (Pa^n s)	n	λ
Sediments	$0.64 + \frac{807}{T+77}$	1.5×10^{-6}	1000	0.150	1	Wet Qz.	1.97×10^{17}	2.3	1.00
Upper cont. crust	$0.64 + \frac{807}{T+77}$	1.00×10^{-6}	1000	0.150	1	Wet Qz.	1.97×10^{17}	2.3	1.00
Lower cont. crust	$1.18 + \frac{474}{T+77}$	0.25×10^{-6}	1000	0.150	1	Pl. (An75)	4.80×10^{22}	3.2	1.00
Upper oceanic crust	$0.64 + \frac{807}{T+77}$	0.25×10^{-6}	1000	0.000	1	Wet Qz.	1.97×10^{17}	2.3	1.00
Lower oceanic crust	$1.18 + \frac{474}{T+77}$	0.25×10^{-6}	1000	0.600	1	Pl. (An75)	4.80×10^{22}	3.2	1.00
Low Velocity Zone	$0.73 + \frac{1293}{T+77}$	2.20×10^{-8}	1000	0.600	1	Dry Ol.	$3.98 \times 10^{18*}$	3.5	1.00
Mantle	$0.73 + \frac{1293}{T+77}$	2.20×10^{-8}	1000	0.600	1	Dry Ol.	3.98×10^{16}	3.5	1.00
Weak Zone	$0.73 + \frac{1293}{T+77}$	2.20×10^{-8}	1000	0.000	1	Wet Ol.	5.01×10^{20}	4.0	1.00

* $\eta_0 = \eta_{eff}$

Supplementary Table S1 – Rheological[37] and thermal[57] parameters of materials used for the experiments.

Model	V_a (J/bar)	α (1/K)	Low Velocity Zone	Mantle Flow Direction	Mantle Flow Velocity (cm/yr)	Slab Dip Angle (°)	Back-arc spreading	Oceanic plate age (Myr)
**a**	1.20	2.00×10^{-5}	Yes	Negative	3.00	74	Yes	20
1	1.40	2.00×10^{-5}	Yes	Negative	3.00	56	Yes	20
2	1.40	3.00×10^{-5}	Yes	Negative	3.00	59	Yes	20
3	1.20	1.00×10^{-5}	Yes	Negative	3.00	63	Yes	20
4	1.40	1.00×10^{-5}	Yes	Negative	3.00	74	Yes	20
6	1.20	3.00×10^{-5}	Yes	Negative	3.00	59	Yes	40
**7**	1.20	2.00×10^{-5}	Yes	Negative	1.00	45	Yes	40
**b**	1.20	2.00×10^{-5}	Yes	Positive	3.00	17.5	No	40
8	1.20	3.00×10^{-5}	Yes	Positive	3.00	20.5	No	20
9	0.80	2.00×10^{-5}	Yes	Positive	3.00	30	No	20
10	1.40	2.00×10^{-5}	Yes	Positive	3.00	17.5	No	20
11	1.40	3.00×10^{-5}	Yes	Positive	3.00	17.5	No	20
**13**	1.20	3.00×10^{-5}	Yes	Positive	3.00	35.5	No	40
14	1.00	3.00×10^{-5}	Yes	Positive	3.00	31	No	40
**Ref.1**	1.00	3.00×10^{-5}	No	Negative	3.00	78.5	Yes	40
**Ref.2**	1.20	2.00×10^{-5}	No	Positive	3.00	17.5	No	40
**Ref.3**	0.80	3.00×10^{-5}	Yes	Null	0.00	67	No	150
**Ref.4**	0.80	3.00×10^{-5}	No	Null	0.00	52.2	No	150

Supplementary Table S2 – Conditions and results of 2D numerical models. Slabs dip in the range of 40°-90° were found for W-directed slabs, while the dip found for E- or SE-directed slabs was within the range of 15°-40°. In bold the reference models commented in the text.

References:

53. Doglioni, C. Foredeeps versus subduction zones. *Geology*, **22**, 271-274 (1994).

54. Carminati, E. & Doglioni, C. Alps vs. Apennines: The paradigm of a tectonically asymmetric Earth. *Earth Sci. Rev.*, **112**, 67-96 (2012).

55. T. Gerya. Introduction to Numerical Geodynamic Modelling. Cambridge University Press, (2010).

56. Faccenda, M., & Dal Zilio, L. (2017). The role of solid–solid phase transitions in mantle convection. Lithos, 268, 198-224.

57. Clauser, C., Huenges, E. Thermal conductivity of rocks and minerals. In: Ahrens, T.J. (Ed.), Rock Physics and Phase Relations. AGU, AGU reference shelf 3, Washington DC, pp. 105–126 (1995).

58. Turcotte, D. L. & Schubert, G. Geodynamics. *3rd ed. Cambridge University Press* (2014).

59. Gerya, T. V., Connolly, J. A. D. & Yuen, D. A. Why is terrestrial subduction one-sided? *Geology*, **36**, 43-46 (2008).

60. Faccenda, M., Gerya, T. V. & Burlini, L. Deep slab hydration induced by bending-related variations in tectonic pressure. *Nat. Geosci.*, **2**, 790-793 (2009).

61. Doglioni, C., Ismail-Zadeh, A., Panza, G. & Riguzzi, F. Lithosphere-asthenosphere viscosity contrast and decoupling. *Phys. Earth Planet. Inter.*, **189**, 1-8 (2011).

62. Crameri, F., Schmeling, H., Golabek, G. J., Duretz, T., Orendt, R., Buiter, S. J. H., & Tackley, P. J. A comparison of numerical surface topography calculations in geodynamic modelling: an evaluation of the 'sticky air' method. Geophysical Journal International, 189(1), 38-54 (2012).

63. Dal Zilio, L., Faccenda, M., and Capitanio, F. The role of deep subduction in supercontinent breakup. *Tectonophysics* (2017).
